# Rapid, random‐access, and quantification of hepatitis B virus using the Cepheid Xpert HBV viral load assay

**DOI:** 10.1002/jmv.26392

**Published:** 2020-08-25

**Authors:** Ali M. Auzin, Serena Slavenburg, Cas Peters, Greet Boland, Janette Rahamat‐Langendoen, Willem J.G. Melchers, Rob Schuurman

**Affiliations:** ^1^ Department of Medical Microbiology Radboud University Medical Center Nijmegen The Netherlands; ^2^ Department of Medical Microbiology University Medical Center Utrecht Utrecht The Netherlands

**Keywords:** hepatitis B virus, molecular diagnostics, random‐access testing, viral load

## Abstract

**Background:**

Monitoring viral load (VL) is an essential part of the management of patients chronically infected with hepatitis B virus (HBV). The commercial HBV VL assays currently available are generally performed on high‐throughput platforms for batch wise testing of plasma samples, with relatively long turn‐around‐times. Rapid VL testing could provide immediate input to clinical decision making.

**Methods:**

One hundred two stored plasma samples from 102 patients who were previously tested for HBV VL by the Cobas Ampliprep/Taqman or Cobas 4800 (Roche, Pleasanton, CA), were analyzed by the recently introduced Cepheid Xpert HBV Viral Load Assay. Thirty‐one of the 102 samples were negative for HBV DNA and 71 out of 102 samples had a detectable VL. HBV DNA loads ranged from <20 to 5E8 IU/mL. HBV genotypes (A, B, C, D, E, and G) were known for 52 of the VL positive samples. Correlation of VL results between both assays was determined by the Pearson correlation coefficient (*r* ^2^). The level of concordance was assessed using the Bland‐Altman analysis.

**Results:**

HBV VLs correlated well between both assays, across all genotypes (Pearson correlation coefficient *r* ^2^ = 0.987). Six samples exceeded a 0.5  log difference between assays. Bland‐Altman analysis demonstrated a mean of the difference of −0.107 log and a standard deviation of 0.271 log.

**Conclusion:**

High correlation was observed between the Roche Cobas HBV Viral Load tests and the Xpert HBV Viral Load Assay, thus enabling rapid, random access, and accurate HBV VL assessment.

## Highlights

‐ Comparison of viral load testing between Cobas Ampliprep/Taqman/4800 and the Xpert HBV Viral Load Assay.

‐ High correlation observed between Roche Cobas HBV viral load test and Xpert HBV Viral Load Assay.

## 1 BACKGROUND

Infections with hepatitis B virus (HBV) are a global health care burden. It is estimated that around two billion individuals worldwide have evidence of past or present infection with HBV. The estimated number of people with chronic hepatitis B virus (CHB) infections, defined by positive HBsAg, is 257 million.^1^ Approximately 700 000 deaths occurred globally in 2015 due to cirrhosis and 470 000 deaths due to primary hepatocellular carcinoma as a result of HBV infections.^1^

Accurate measurement of HBV viral load (VL) is important since the VL is an important part of current guidelines for the management of CHB. Treatment initiation is based on extent of liver disease in combination with the magnitude of the VL. Infrequent VL testing or failure to do so, may lead to ongoing suboptimal treatment with a risk of resistance development or accumulation against antiviral agents and long‐term risks on liver cirrhosis and hepatocellular carcinoma. VL monitoring is also crucial during pregnancy, where a high VL is a criterium to start antiviral treatment to prevent mother to child transmission, and in immunocompromised patients at risk of HBV reactivation.^2^, ^3^, ^4^, ^5^, ^6^

Current HBV viral load assays are usually performed on high‐throughput platforms for batch wise testing of plasma samples. Rapid VL testing using a random‐access system could provide quick results to support clinical decision making. In this study HBV VL assessment using the Cobas Ampliprep/Taqman (CAP/CTM) or Cobas 4800 (C4800) (Roche, Pleasanton, CA) was compared with results generated using the new Cepheid HBV Viral Load Assay on the GeneXpert instrument System (Cepheid, Sunnyvale, CA).

## 2 MATERIALS AND METHODS

### 2.1 Samples

Stored EDTA‐plasma samples of 106 patients from the Radboud University Medical Center (RUMC) Nijmegen (n = 64) and the University Medical Center Utrecht (UMCU) (n = 42) were selected based on HBV VL results as determined by the routine laboratory tests (CAP‐CTM for UMCU and C4800 for RUMC). Thirty‐two of 106 samples were included that had previously been tested negative for HBV DNA. For 54 out of 106 samples the HBV genotype was known.

Plasma samples were tested either at RUMC or at UMCU and test results were combined, no operator bias was detected between the two centers. For independent external quality assessment purposes, theHBVDNA18 EQA panel (quality control for molecular diagnostics [QCMD], Glasgow UK), consisting of eight samples, was tested on both assay platforms at RUMC.

### 2.2 Laboratory analysis

The Xpert HBV Viral Load Assay on the GeneXpert random access system is a cartridge based, quantitative molecular test with a limit of quantification of 10 IU/mL and a detection limit of 3.2 IU/mL in plasma, and with a run time of approximately 1 hour and 30 minutes. For the CAP‐CTM or C4800, the limit of quantification was 20 IU/mL and the limit of detection was 4.4 IU/mL, with a turnaround time of approximately 6 hours, from the moment the test was started. The input volume for the Xpert HBV Viral Load Assay was 600 µL, the input for the CAP‐CTM/C4800 was 400 µL.

### 2.3 Data analysis

The results obtained by both tests were translated from HBV DNA IU/mL to log IU/mL for further statistical analysis. In positive samples where only one of the measurements fell outside of the measurement range, the measurement was set at the lowest or highest measurable point (eg, (CAP/CTM/C4800 %3E1.7E8 IU/mL becomes 1.7E8 IU/mL and <20 IU/mL becomes 20 IU/mL for data analysis purposes. The differences in VL between the assays were expressed as log difference. The acceptance criteria for inter‐assay differences were set at 0.5 log. The correlation of the results of both assays was determined by calculating the Pearson correlation coefficient (*r* ^*2*^). The level of concordance was assessed using Bland‐Altman analysis. The mean of the differences between the two tests was calculated and plotted against the mean of the measurements. The mean and the standard deviation (SD) of all log translated VL results were calculated using a one‐sample *t* test. The 95% limits of agreement were determined as the mean ± 1.96 SD.

All calculations were done using SPSS statistics version 25 (IBM, New York).

## 3 RESULTS

A total 102 samples from the original 106 samples were tested in this study. Three cartridge errors and three sample errors occurred, resulting in four samples that could not be repeated due to insufficient material and were therefore not included in the analysis. Thirty‐one samples previously tested negative on the CAP/CTM or C4800 for HBV DNA. Two of these samples had a VL of <10 IU/mL on the GeneXpert, the remaining samples tested negative in both assays. Seventy‐one of 102 samples had a detectable VL result. Genotypes were known for 52 out of 71 samples and included genotype A (n = 14), genotype B (n = 5), genotype C (n = 8), D (n = 21), E (n = 3), and G (n = 1). Genotypes were unknown for the remaining 19 samples with a detectable VL result.

**Figure 1:**
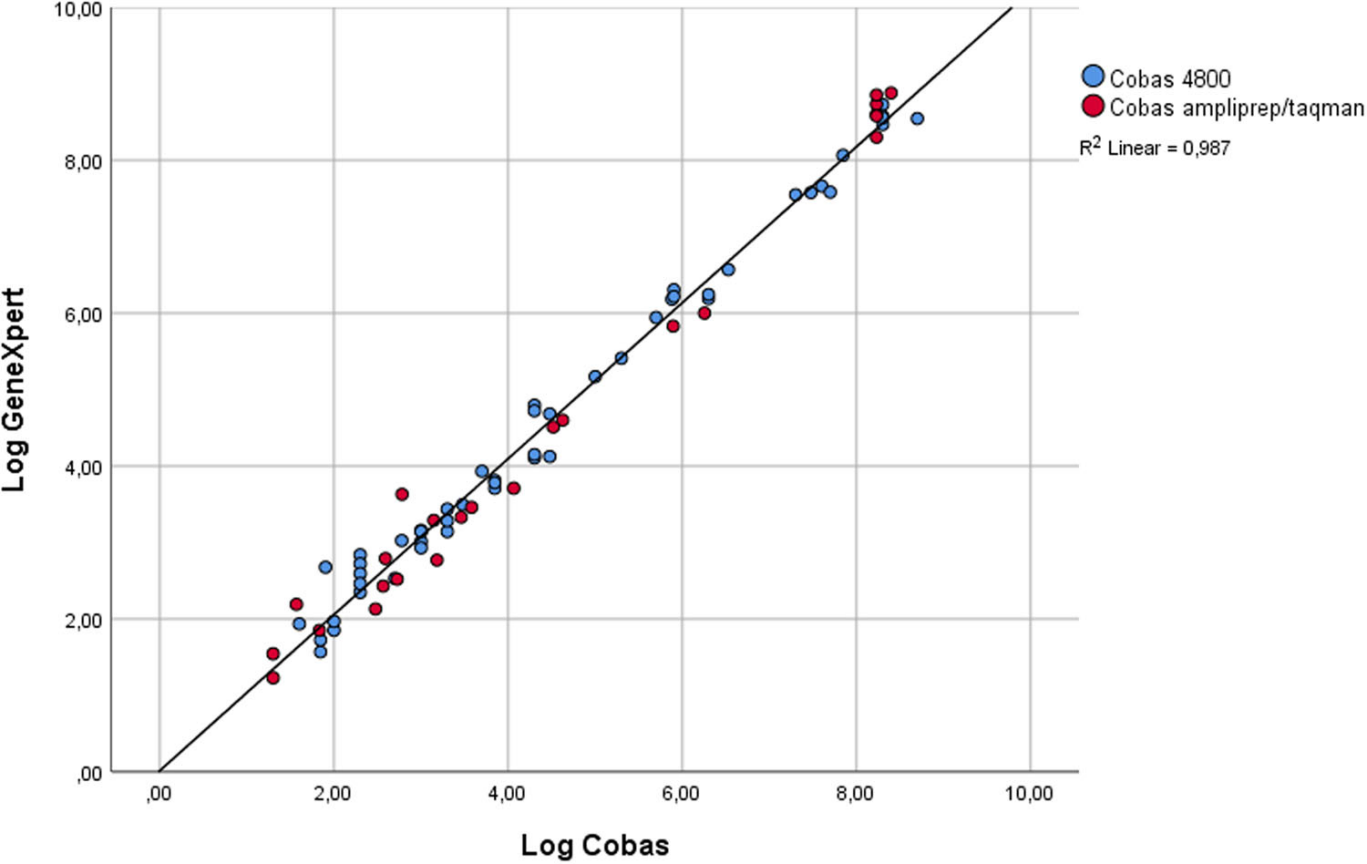
Correlation curve for the GeneXpert versus the Cobas ampliprep/taqman/4800

High correlation of HBV VL across all tested genotypes were found between the HBV viral load assays (Figure 1, Pearson correlation coefficient *r* ^*2*^ = 0.987). The maximum log difference between the two tests was 0.85 log, the minimum log difference was 0.01 log.

Six samples exceeded a 0.5 log difference. Four of these samples all had low VLs in CAP‐CTM and C4800 assays and ranged between 37 and 605 IU/mL. VLs of these samples for the Xpert HBV Viral Load assay were all higher and ranged between 154 and 4300 IU/mL. The other two samples were on the other end of the spectrum, with VLs that exceeded the highest measurable VL on the CAP‐CTM and C4800 assays (%3E1.7E8 IU/mL). Similarly, the VLs were higher on the Xpert HBV Viral Load assay, respectively 5.4E8 and 7.1E8 IU/mL.

**Figure 2:**
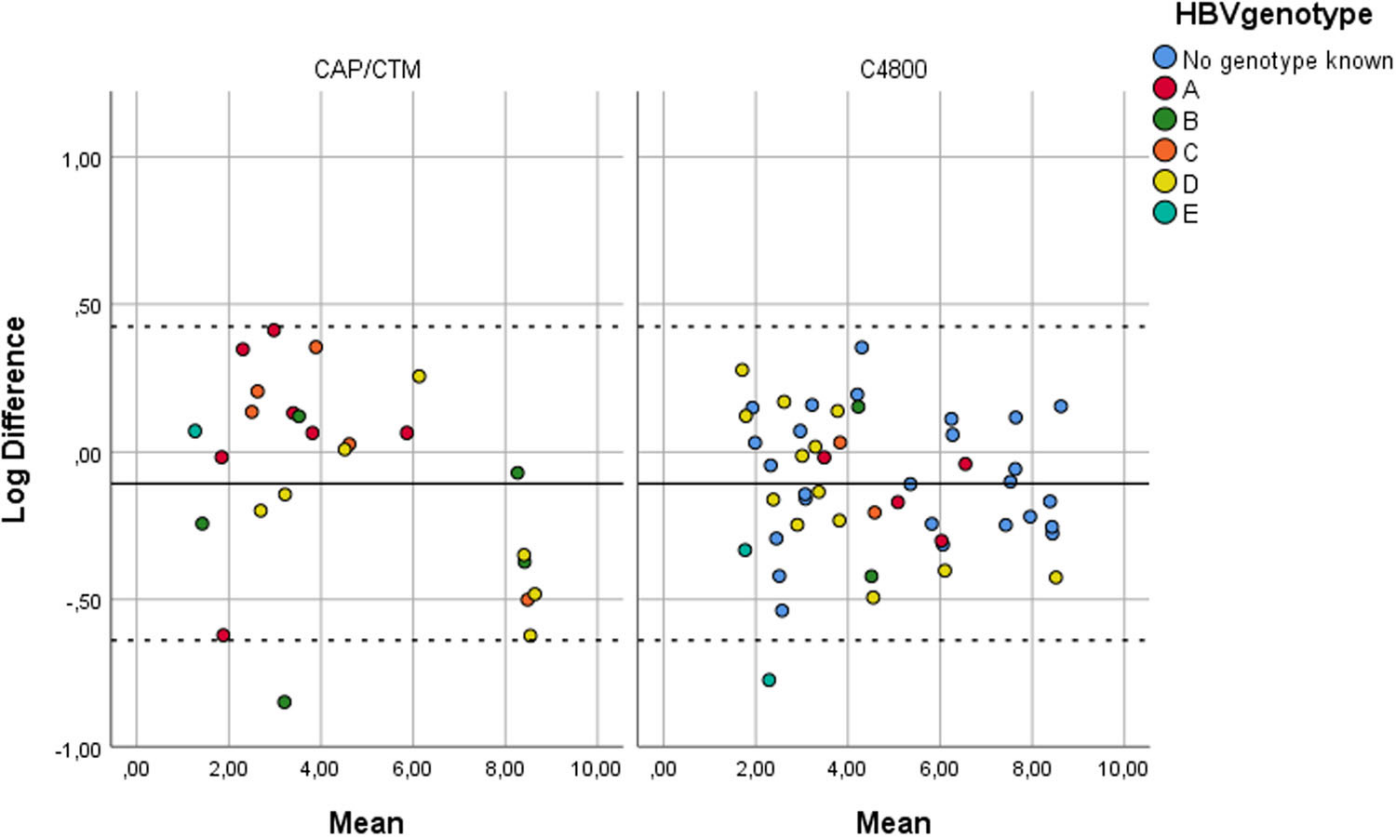
Bland‐Altman plot for Cobas ampliprep/taqman/4800 versus GeneXpert. The solid line marks the mean difference, while the dashed lines mark the limits of agreement

High agreement was seen between the tests in the Bland‐Altman analysis, with a mean of the differences of −0.107 log and a standard deviation of 0.271 log (Figure 2).

Results obtained with QCMD EQA samples tested in the GeneXpert assay demonstrated to meet the consensus results (Table 1). All results for QCMD samples that were run on the GeneXpert were within 0.5 log difference of the consensus result reported by QCMD, the largest difference being 0.210 log.

**Table 1:** Comparison of QCMD samples with Cepheid GeneXpert viral load assay QCMD

QCMD results					
QCMD code	Genexpert		QCMD		
	HBV	HBV	QCMD	Upper	Lower
	load	load	Results	border	border
	IU/mL	Log10	Log10	Log10	Log10
HBVDNA17‐01	5.57E + 03	3.746	3.771	3.023	4.415
HBVDNA17‐02	5.77E + 04	4.761	4.743	3.865	6.438
HBVDNA17‐03	Neg	Neg	Neg	Neg	Neg
HBVDNA17‐04	7.01E + 02	2.846	3.056	2.301	4.585
HBVDNA17‐05	1.02E + 03	3.009	3.055	2.646	3.845
HBVDNA17‐06	6.39E + 02	2.806	2.700	1.785	4.324
HBVDNA17‐07	5.65E + 02	2.752	2.793	1.771	3.643
HBVDNA17‐08	7.29E + 02	2.863	3.066	2.029	4.784

Abbreviation: QCMD, quality control for molecular diagnostics.

## 4 DISCUSSION

Correlation and agreement between the routine laboratory assays (CAP‐CTM and C4800) and the Xpert HBV Viral Load assay was evaluated for 102 samples previously tested on CAP‐CTM or C4800. A high agreement was observed between HBV VL results generated in routine laboratory assays (CAP‐CTM and C4800) and the Xpert HBV Viral Load assay across all tested genotypes.

Four of the samples that exceeded a 0.5 log difference all had low VLs (CAP‐CTM/C4800: 37 605 IU/mL versus GeneXpert: 154 and 4300 IU/mL. The other two samples that exceeded 0.5 log difference can be explained by the fact that these samples exceeded the measurable VL on this assay, %3E1.7E8 IU/mL, on the CAP‐CTM/C4800 and were set at 1.7E8 IU/mL for statistical analysis purposes. Therefore, the true VL is unknown and could in fact lie closer to the VL as found in the Xpert HBV Viral Load assay. The samples that exceeded 0.5 log difference were from both laboratories, which indicates that the discrepancy in VLs is irrespective of the method of testing. The diagnosis and determination of the phase of the infection can in part be based on the measurement of HBV VL. The HBV VL is also used for treatment decisions and monitoring of patients, though in the context of ALT levels and the severity of the liver disease. In addition, adjustment to an existing treatment regimen will usually be based on VL results from to subsequent samples.

External quality assessment, with the use of the HBVDNA18 EQA panel (QCMD, Glasgow UK) demonstrated VL results that corresponded with the known consensus VLs of the samples. This further strengthens the correlation and agreement found between the assays, across all the genotypes tested, as seen in both the bland‐Altman plot and the calculated Pearson correlation coefficient.

The relatively small sample size and the fact that not all genotypes were represented in this evaluation, are limitations of this study. Most of the samples contained HBV genotypes A, C, or D. The genotypes previously found in the Netherlands include A, B, C, D, E, F, G,^7^ the most prevalent of which are A and D.^8^

Cost effective batchwise testing on high‐throughput systems requires multiple samples to be tested per run. As a result, most laboratories perform such batch runs once or twice a week. This may result in a relatively long window between sample collection and availability of the result. In contrast the random‐access cartridge‐based testing can be done throughout the day whenever a new sample is delivered to the laboratory. The cartridge‐based nature of the test negates the need to use control samples, because each cartridge contains multiple quality control (QC) steps. These QC steps ensure that the volume of the sample that has been added is sufficient and the probes that are present in the cartridge are in order, before the polymerase chain reaction (PCR) reaction is started. The quantification of the HBV DNA concentration in the sample is done by using high and low internal quantitative standards in tandem with specific acceptance criteria. Additionally, these internal quantitative standards are used as controls to detect specimen‐associated inhibition of the PCR reaction.

The pretest preparations take less than 10 minutes. The turnaround time of the Xpert HBV Viral Load assay was approximately an hour. After the test has reached completion, the results need to be checked and confirmed before they can be conveyed to the clinician. Therefore, the total time from the cartridge preparation to the release the VL results, is approximately 1 hour and 30 minutes.

The random‐access nature and fast assay turnaround time of the GeneXpert changes the dynamics and routing of HBV VL testing in the routine microbiological laboratory and leads to a more efficient laboratory workflow and a significant improvement in speed in which the result is available for patient care.

In conclusion high agreement and correlation were observed between both HBV viral load assays with regard to the HBV genotypes tested. The Xpert HBV Viral Load assay is a user‐friendly random‐access test for rapid, accurate virological assessment of HBV infected patients.

## CONFLICT OF INTERESTS

None of the contributing authors have any conflicts of interest, except that the cartridges used in the viral load testing of Hepatitis B virus on the GeneXpert instrument System (Cepheid, Sunnyvale, CA) were provided by Cepheid.

## AUTHOR CONTRIBUTIONS

AMA, SS, and CP performed testing. AMA analyzed the data and wrote the manuscript with input from all authors.

## Data Availability

The data that support the findings of this study are available from the corresponding author upon reasonable request.
